# Coapplication of Chicken Litter Biochar and Urea Only to Improve Nutrients Use Efficiency and Yield of *Oryza sativa* L. Cultivation on a Tropical Acid Soil

**DOI:** 10.1155/2015/943853

**Published:** 2015-07-27

**Authors:** Ali Maru, Osumanu Ahmed Haruna, Walter Charles Primus

**Affiliations:** ^1^Department of Crop Science, Faculty of Agriculture and Food Sciences, Universiti Putra Malaysia, Bintulu Campus, 97008 Bintulu, Sarawak, Malaysia; ^2^Agriculture and Environment, Borneo Eco-Science Research Center, Faculty of Agriculture and Food Sciences, Universiti Putra Malaysia, Bintulu Sarawak Campus, 97008 Bintulu, Sarawak, Malaysia; ^3^Institute of Tropical Forestry and Forest Products (INTROP), Universiti Putra Malaysia, 43400 Serdang, Selangor, Malaysia; ^4^Department of Basic Science and Engineering, Faculty of Agriculture and Food Sciences, Universiti Putra Malaysia, Bintulu Campus, 97008 Bintulu, Sarawak, Malaysia

## Abstract

The excessive use of nitrogen (N) fertilizers in sustaining high rice yields due to N dynamics in tropical acid soils not only is economically unsustainable but also causes environmental pollution. The objective of this study was to coapply biochar and urea to improve soil chemical properties and productivity of rice. Biochar (5 t ha^−1^) and different rates of urea (100%, 75%, 50%, 25%, and 0% of recommended N application) were evaluated in both pot and field trials. Selected soil chemical properties, rice plants growth variables, nutrient use efficiency, and yield were determined using standard procedures. Coapplication of biochar with 100% and 75% urea recommendation rates significantly increased nutrients availability (especially P and K) and their use efficiency in both pot and field trials. These treatments also significantly increased rice growth variables and grain yield. Coapplication of biochar and urea application at 75% of the recommended rate can be used to improve soil chemical properties and productivity and reduce urea use by 25%.

## 1. Introduction

Nitrogen fertilizers use is expected to increase in a stabilized way up to 21.3 million tonnes in 2015 and 23.6 million tonnes by 2030 [1], suggesting that N is an important nutrient in rice cultivation as it plays an essential role in sustaining high yield of crops [2, 3]. This is probably one of the reasons why 70% of the chemical fertilizers used in rice cultivation are N fertilizer. Nitrogen is generally applied to soils in a large quantity [4–6] due to demand of N by high yielding rice cultivars to achieve a desirable yield [7]. Furthermore, there is no residual effect of N in paddy fields [8] because some of the N is immobilized by microbes into soil organic fraction, and some is fixed by the clay minerals such as illite, vermiculite, and smectite whereas the rest are lost through denitrification, ammonia volatilization, and leaching. However, N use can be efficiently managed through the use of biochar to improve N and other important nutrients uptake in rice cultivation [8]. Nutrient uptake by rice plants is not different from monocot crops such as wheat and maize but the amount of nutrients absorbed varies with rice growth stage. Nitrogen absorption is low at seedling stage and peaks before heading stage [9, 10]. The stage of highest P uptake is young panicle developmental stage followed by the tillering stage [9, 10]. The period of highest K uptake is before heading stage and little is absorbed after heading [11–14]. Nutrient absorption differs with rice cultivar, fertilizer type, fertilization technology, soil type, and environmental factors [15–18]. The soil (Typic Paleudults) used in this study is less cultivated with rice compared to Alfisols, Vertisols, Mollisols, and Inceptisols due to its poor physical and chemical properties [19]. However, Ultisols are the most common agricultural soils in the tropics. Biochar can be used to improve the physicochemical properties of Ultisols to boast rice yield on these soils.

Biochar is pyrolysis biomass under limited or no supply of oxygen [20]. Biochar has an impact on nutrient addition and nutrient retention in soils. Biochar consists mainly of mineral elements such as Ca, Fe, Mg, Na, K, P, Si, and Al [21] with minimum amount of N. During pyrolysis, significant proportions of biomass N are lost by volatilization [22]. The N remaining in the biochar and the fraction of N inside aromatic C structures of biochar tend to be poorly available for plants uptake [22, 23]. Biochar has a low density and high porosity that makes it possible to inhabit soil microorganisms and hold moisture up to three times its own weight [24] thereby preventing nutrient leaching and volatilization. Surface water infiltration is improved in a biochar amended soil [25–27]. Biochar consists largely of amorphous graphene sheets, which give rise to large amounts of reactive surfaces where a wide variety of organic (both polar and nonpolar) molecules and inorganic ions are absorbed [28] and made available for plants absorption. High pH of biochar increased acidic soil pH [29]. An increase in pH provides a wide range of benefits in terms of soil quality, notably by improving the availability of nutrients to plants, and in some cases it reduces the availability of detrimental elements such as Al and Fe [29]. The objectives of this study were to (i) increase rice yield through the use of biochar and N fertilizer only and (ii) reduce N fertilizer application rate by improving nutrients use efficiency. These objectives were based on the assumptions that chicken litter biochar used in this study will provide all essential nutrients recommended for rice production except N and it will also release and enhance efficient use of P and K in the soil for rice plant growth.

## 2. Materials and Methods

Typic Paleudults (Nyalau Series) soil was sampled at the 0 to 25 cm depth in an uncultivated secondary forest of Universiti Putra Malaysia, Bintulu Campus, Sarawak, Malaysia (latitude 3° 12′ 14.5′′ N and longitude 113° 4′ 16.0′′ E). The soil was air-dried after which it was ground to pass a 5 mm sieve for pot trial and further sieved to pass a 2 mm sieve for analysis of selected chemical and physical properties of the soil before and after the pot and field experiments. Soil pH was determined in 1 : 2.5 (soil : distilled water) using a digital pH meter [30]. Soil organic matter was determined using loss of weight on ignition after which the total carbon was calculated as 58% of the organic matter [31]. Total N was determined using Kjeldahl method [32] and inorganic N (NO_3_^−^-N and NH_4_^+^-N) was determined using the method described by Keeney and Nelson [33] whereas total P was determined using UV-Vis Spectrophotometer (Perkin Elmer Lambda 25, USA) after blue color was developed according to the Blue method [34]. Exchangeable cations were extracted with 1 M NH_4_OAc, pH 7.0 using the leaching method [35], and determined using Atomic Absorption Spectrometer (AAnalyst 800, PERKIN Elmer Instruments, Norwalk, CT). The soil cation exchange capacity (CEC) was determined with a leaching method [35] followed by steam distillation [36].

### 2.1. Chemical Composition of Biochar

The Black Earth Products chicken litter biochar used in this study was imported from Australia. The chemical properties (Table 1) of the biochar were up to standard whereas the arsenic, cadmium, chromium, copper, lead, mercury, nickel, and zinc levels are all below the set guidelines for maximum levels of heavy metals (20, 5, 250, 375, 150, 4, 125, and 700 mg Kg^−1^, resp.) based on Australia Certified Organic Standard, 2010 (Table 1).

### 2.2. Pot Study

In the greenhouse study, pots (864.33 cm^3^) were filled with 1 kg of air-dried soil (based on the bulk density of the soil that is 1.157 g cm^−3^) that was mixed thoroughly with 20 g of the chicken litter biochar. The four replicates of each treatment were arranged in a basin and the basins were arranged in a rain shelter at Universiti Putra Malaysia, Bintulu Sarawak Campus, in a Complete Randomized Design (CRD). 15-day nursed rice seeds of MR219 variety in a plastic-ware prior to transplanting were planted at a planting density of 3 seedlings per pot.

Treatments evaluated are as follows:

- soil only (T1),
- soil + normal fertilization (T2),
- soil + biochar + normal fertilization (T3),
- soil + biochar + 100% N fertilization only (T4),
- soil + biochar + 75% N fertilization only (T5),
- soil + biochar + 50% N fertilization only (T6),
- soil + biochar + 25% N fertilization only (T7),
- soil + biochar only (no fertilization) (T8).

The fertilizers used for the MR219 variety are the recommended fertilizer rates for rice by Muda Agricultural Development Authority (MADA), Malaysia [37] (Table 2).

The recommended rates (Table 2) by MADA [37] were scaled down based on the requirement of plant hill and the various percentages of N used for pot study (Table 3).

The water level in the basins was maintained at 2.5 cm above the soil in the pot to mimic waterlogged condition. The fertilizers were applied on the soil surface in each pot at the growth stages recommended by MADA [38] (Table 3). However, all plants under N fertilization only show K deficiency at 35 days after transplanting and to correct this deficiency, 0.24 g hill^−1^ MOP was applied. The plants were managed and harvested at panicle heading stage (70th day after transplanting) which is a major determinant of rice yield [39]. Plant height, number of tillers, and number of leaves were measured at 70 days after transplanting before harvesting the above biomass for dry matter yield and chemical analysis. The soil in the pots was air-dried and ground to pass a 2 mm sieve for analysis. The soil samples were analyzed using the standard procedures stated previously. The rice plant roots were thoroughly washed with tap water followed by distilled water after which they were oven-dried for dry weight and chemical analysis. The roots and the above biomass samples were digested using the Single Dry Ashing Method [35] after which K, Ca, Mg, Mn, Zn, Fe, and Cu were determined using Atomic Absorption Spectrometry (AAS) whereas P was determined using the Blue method [36]. Total N was determined using Kjedahl method [32]. Crude silica was also determined using the method described by Shouichi et al. [38]. The nutrient concentrations were multiplied by their dry matter yield to represent nutrient uptake. The agronomic and crop recovery efficiency of applied N was determined using the formula below:

(1) AEN=YN−Y0FN,REN=UN−U0FN,

where *F*_N_ is amount of (fertilizer) N applied (kg ha^−1^), *Y*_N_ is crop yield with applied N (kg ha^−1^), *Y*_0_ is crop yield (kg ha^−1^) in a control treatment with no N, *U*_N_ is total plant N uptake in aboveground biomass at maturity (kg ha^−1^) in a plot that received N, and *U*_0_ is the total N uptake in aboveground biomass at maturity (kg ha^−1^) in a plot that received no N [40].

### 2.3. Field Study

A field study was conducted after the pot trial at the Long Term Research Grant Scheme (LRGS) rice plot at Universiti Putra Malaysia Bintulu campus on the same type of soil (Typic Paleudults) used in the pot experiment. The experimental area has an annual precipitation of 2,200 mm and a maximum and minimum mean temperature of 32 and 24°C, respectively. The study area also has a relative humidity of 70 and 90%. The experimental design used was randomized complete block design with four replications (blocks). The total experimental area was 24 m (length) × 23 m (breadth). Each plot size was 2 m (length) × 2 m (breadth). The distance between plots was 1 m and that between blocks was 3 m. The soil pH, P, K, Cu, Zn, Ca, Fe, and Mg and total N, NO_3_^−^, and NH_4_^+^ of the experimental plots were determined before and after the study using the procedures described previously in the pot trial. The treatments evaluated in this field study were the same as those in the pot study except T3 (soil + biochar + normal fertilization) which was excluded. T3 was excluded in this field trial because its effect on dry matter production in the pot trial was not statistically different from those of T4 and T5 (Table 7). The biochar and the fertilizer rates used in the pot study (Table 3) were scaled up in the field experiment (Table 4).

The biochar was spread on the soil surface of the experimental plots and thoroughly mixed a day before transplanting. 15-day nursed rice seeds of MR219 variety in a plastic-ware prior to transplanting were planted at a planting density of 100 hills per experimental plot and 3 seedlings per hill with a planting distance of 0.2 m between rows and 0.2 m within. The water level in the experimental plot was maintained about 4 cm above the soil surface to mimic waterlogged condition. The rice plants were managed and harvested at different maturity day due to treatments effect on grain ripening. Plant height, number of tillers, number of leaves, culm height, and number of panicles were measured at maturity (a day before harvesting the above biomass) for dry matter yield and chemical analysis. Ten panicles were collected from each experimental plot for grain filling and yield determination. The soils were collected from the experimental plots, air-dried, and ground to pass a 2 mm sieve. The soil and above biomass samples were analyzed using the standard procedures stated in the pot study.

### 2.4. Statistical Analysis

Analysis of variance (ANOVA) was used to test treatment effects whereas treatments means were compared using Tukey's test [41]. Simple linear regression and Pearson correlation were used to establish relationship between variables. The Statistical Analysis Software version 9.3 was used for the statistical analysis.

## 3. Results and Discussion

### 3.1. Effects of Biochar and N Rates on Soil Chemical Properties

The pH of the soil with coapplication of biochar and urea only (T3, T4, T5, T6, T7, and T8) of the pot trial were significantly higher than that in the normal fertilization (T2) and soil only (T1) (Table 5). The exchangeable acidity and Al^3+^ of the soil with coapplication of biochar and urea only (T3, T4, T5, T6, T7, and T8) in the pot trial were statistically lower than in T2 and T1 whereas H^+^ in T5, T6, T7, and T8 were lower than in T2 (Table 5). These differences were due to application of biochar as biochar has high affinity for these ions. In the field trial, Al^3+^ in T4, T5, T6, and T7 were significantly lower than in T2 and T1. However, the pH, exchangeable acidity, and H^+^ of the soil due to T3, T4, T5, T6, T7, and T8 in the field trial were not statistically different from those of T2 and T1 (Table 6) because of the large volume of soil in the field (in terms of ratio to the amount of biochar used), hence reducing the effect of biochar compared to the specific amount of soil used in the pot trial. It might also be due to high acidic cations such as H^+^ in the field which might have caused buffer changes in active acidity. Although the pH, exchangeable acidity, and H^+^ of the soil with biochar (T3, T4, T5, T6, T7, and T8) in the field trial were not remarkably reduced, the reduction of Al^3+^ can be considered as the reduction of the soil acidity as Kong et al. [6] proposed that reduction of aluminum toxicity in tropical soils leads to reduction of soil acidity and this process improves plant productivity. In the pot trial, the effects of T2, T3, T4, T5, T7, and T8 on OM, TC, Mn^2+^, Fe^2+^, Zn^2+^, Na^+^, Ca^2+^, Mg^2+^, NO_3_^−^, NH_4_^+^, total N, CEC, and K^+^ were similar. However, Cu^2+^, total P, and available P were significantly higher in T3, T4, T5, T6, T7, and T8 than in T2 (Table 5). In the field trial, CEC, OM, and TC in T4, T5, T6, and T7 were statistically higher than in T2 and T1 but NH_4_^+^ was significantly higher in T5, T6, and T7 than in T2 and T1 (Table 6). The soil NH_4_^+^, OM, and TC in the field were increased due to biochar application [29]. Additionally, total N and available P of the plots which received T5 and T6 in the field trial were significantly higher than in T2 and T1. However, the effects of T2, T3, T4, T5, T6, T7, and T8 on soil Cu^2+^, Mn^2+^, Zn^2+^, Na^+^, Ca^2+^, Mg^2+^, NO_3_^−^, total P, total K^+^, and exchangeable K^+^ were similar (Table 6). Although Nyalau Series is not productive and also prone to nutrient leaching under flooded condition [42], the chicken litter biochar used in this study generally improved the chemical properties of this soil [43]. The differences in some of the chemical elements among the soils amended with biochar were due to substitution between different nutrient elements in the rice plants [44]. Furthermore, the nitrogen rates (100%, 75%, 50%, 25%, and 0%) in T4, T5, T6, T7, and T8 stimulated the availability of other nutrients especially available P and K (Tables 5 and 6).

### 3.2. Aboveground Variables

In the pot study, plant height, number of leaves, number of tillers, and dry matter yield (DMY) due to T3, T4, and T5 were significantly higher than in T2 and T1. However, plant height, number of leaves, and dry matter yield (DMY) among T3, T4, and T5 were not significantly different but the number of tillers was not significantly different between T3 and T4 (Table 7). In the field study, number of tillers and plant height due to T2 and T1 were not significantly different from those of T4, T5, and T6 (Table 8). However, culm height due to T4, T5, T6, T7, and T8 was significantly lower than in T2 and T1. The number of leaves in T4, T5, T6, and T7 was significantly higher than in T2 and T1 (Table 8). The number of panicles in T4 and T5 was higher and significantly different from those of T2 and T1 (Table 8). The differences in nutrients availability in the soil (Tables 5 and 6) due to coapplication of biochar and urea only might have caused the differences in the aforementioned growth variables, confirming the findings of Brady and Weil [29] that biochar improves soil productivity and N plays an important role in sustaining high yield of rice [2, 3]. The percentage of total grain filling was not statistically different in all the treatments; however the total grain and dry matter yield in T2 was statistically lower than in T4 and T5 (Table 8). The grain yield in T5 and T4 was significantly higher than in T2 and T1 (Table 8). The differences in number of panicles due to the effect of biochar on nutrient availability and nutrient use efficiency of N fertilization might have caused the differences in the grain yield, total grain, and dry matter yield (Tables 7 and 8). The grain yields of T4 and T5 were not significantly different although T4 had 100% N fertilization, that is, 25% more than in T5 (Table 8). This indicates that biochar can be used to reduce N application rate in paddy cultivation on tropical acid soils. The yield of T5 (7.556 t ha^−1^) was 44.36% higher than that of T2 (4.206 t ha^−1^) (Table 8). Leaching of soil nutrients due to coarse particles in the soil of this present study might have reduced the number of tillers bearing grains of the plants under T2 and hence the lower yield. This confirms the findings of McLaughlin et al. [24] that biochar reduces leaching of nutrients. The rice yields of T5 and T4 were lower than the potential yield of about 10 t ha^−1^ due to limitation of some nutrients especially P and K. Although T4 and T5 had limited P and K, their yields are higher than the average rice yield of 4 to 5 t ha^−1^ in Malaysia (Table 8).

### 3.3. Nutrient Uptake

The effects of biochar and N fertilization on nutrient uptake of the rice plants in both pot and field trials were determined (Tables 9 and 10). The pot trial shows that Ca^2+^, K^+^, Mg^2+^, Cu^2+^, and Mn^2+^ uptake were statistically lower in T2 than in T3, T4, T5, and T6 (Table 9). However, Zn^2+^, total N, and crude silica due to T2 were not significantly different from those of T3, T4, T5, and T6 (Table 8). The uptake of Fe^2+^ was significantly higher in T2 than in T3, T4, T5, T6, T7, and T8 whereas total P of T3 and Na^+^ of T5 uptake were higher and statistically different from those of T2 (Table 9). In the field trial, Mg^2+^ and total P uptake in T4, T5, and T6 were significantly higher than in T2 but Ca^2+^, K^+^, and total N uptake in T2 were significantly lower than in T4 and T5 (Table 10). The uptake of Fe^2+^ in T4 was significantly higher than in T2 (Table 10). The difference in Fe^2+^ is due to the higher dry matter yield in T4 as compared to that of T2. The uptake of Na^+^, Cu^2+^, Mn^2+^, and crude silica in T4, T5, T6, T7, and T8 was not statistically different from that of T2 (Table 10), suggesting that the biochar improved both nutrient availability and uptake. Although N uptake in the pot study was higher than the field trial, this difference is because the plants in the pot trial were harvested at panicle initiation stage, a stage where N was not translocated into the sink organs for grain formation compared to the field trial where, at maturity, N was translocated to the sink organs for grain formation. Additionally, some of the urea-N might have been lost through leaching and volatilization in the field trial compared to the pot study. Coapplication of biochar and urea stimulated the availability of other nutrients especially available P and K. Potassium availability was increased by the biochar and urea application due to K^+^ displacement from soil exchangeable complex by the NH_4_^+^ (from urea) confirming the findings of Patrick et al. [45]. Additionally, soluble K^+^ believed to remain at a constant level under flooded condition [45] could not be ascertained because in this study the demand for K by the rice plants exceeded the supplied K in the soil solution at 35 days after transplanting or the soluble K^+^ could not remain at a constant level under flooded condition during the growing period. However, K fertilization was reduced by 62.5% of the recommended K fertilizer by MADA [37].

### 3.4. Relationship between Level of Nitrogen Applied on a Soil Amended with Biochar and Grain Yield

**Figure 1:**
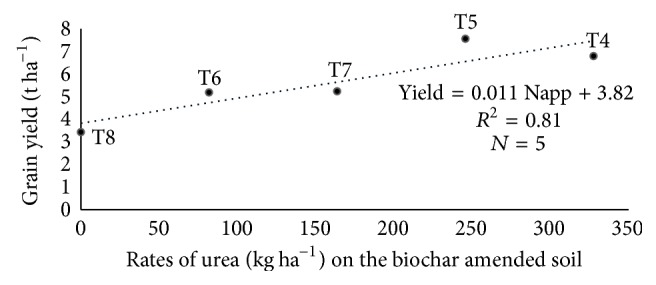
Linear relationship between levels of nitrogen applied on a soil amended with biochar and grain yield.

The relationship between coapplication of biochar and urea (T4, T5, T6, T7, and T8) and rice grain yield was linear (Figure 1), suggesting that grain yield increased with increasing rate of urea.

### 3.5. Correlation among N Fertilization, N, P, and K Uptake, and Grain Yield

Although the relationship between N fertilization and grain yield was linear (Figure 1), it must be noted that the linear relationship in Figure 1 was based on N fertilization only in soils amended with biochar (T4, T5, T6, T7, and T8) and grain yield, whereas the data in Table 11 were obtained based on correlation among N fertilization (T1, T2, T4, T5, T6, T7, and T8), N, P, and K uptake, and grain yield. The linear relationship between urea applied on the soils amended with biochar and grain yield was compared to the correlation between urea applied in all treatments of the study and grain yield. The correlation between N, P, and K uptake and rice grain yield was similar to those of the regression analysis results in Figure 1. However, there was no significant correlation between N fertilization (T1, T2, T4, T5, T6, T7, and T8) and grain yield (Table 11). This contradicted the regression results in Figure 1 where there was significant and positive linear relationship. These results suggest that the biochar increased utilization of urea which resulted in improved grain yield. It is also essential to look at the relationship between nutrient uptake and grain yield instead of focusing only on fertilization and grain yield because the relationship between fertilization and grain yield is influenced by the type of soil on which fertilizers are applied.

### 3.6. Relationship between Internal Nutrient Use Efficiency and Yield

**Figure 2:**
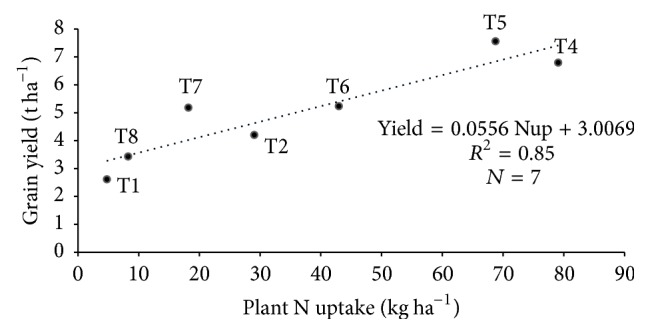
Relationship between N uptake and grain yield under different treatments, where Nup = nitrogen uptake.

**Figure 3:**
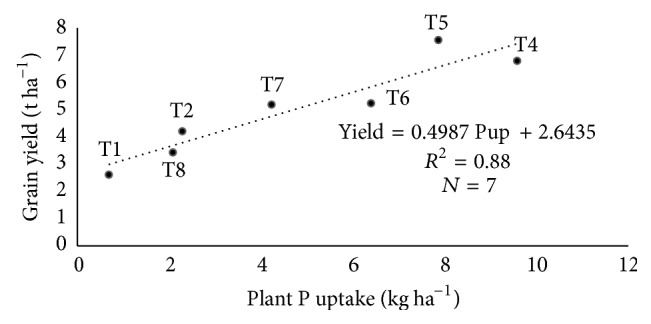
Relationship between P uptake and grain yield under different treatments, where Pup = phosphorus uptake.

**Figure 4:**
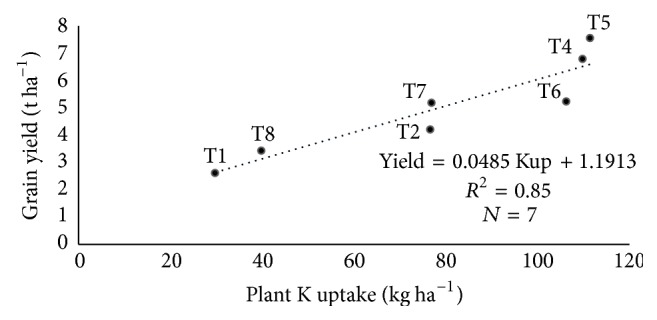
Relationship between K^+^ uptake and grain yield under different treatments, where Kup = potassium uptake.

The internal nutrient efficiency of the major nutrients uptake in response to yield was determined. The aboveground plant N, P, and K uptake in T1 (soil only) were 4.8 kg N ha^−1^, 0.68 kg P ha^−1^, and 29.7 kg K^+^ ha^−1^, respectively, with an average estimated grain yield of 2.61 t ha^−1^ (Figures 2, 3, and 4) whereas aboveground plant N, P, and K uptake in T2 (normal fertilization) were 29 kg N ha^−1^, 2.29 kg P ha^−1^, and 76.6 kg K^+^ ha^−1^, respectively, with an average estimated grain yield of 5.2 t ha^−1^ (Figures 2, 3, and 4). However, the aboveground plant N, P, and K uptake in T5 (soils amended with biochar and 75% urea) were 68 kg N ha^−1^, 7.86 kg P ha^−1^, and 111.5 kg K^+^ ha^−1^, respectively, with an average estimated grain yield of 7.56 t ha^−1^ (Figures 2, 3, and 4) whereas aboveground plant N, P, and K uptake in T5 (soils amended with biochar and 100% urea) were 79.1 kg N ha^−1^, 9.58 kg P ha^−1^, and 109.8 kg K^+^ ha^−1^, respectively, with an average estimated grain yield of 6.79 t ha^−1^ (Figures 2, 3, and 4). Generally, there is a significant relationship between internal nutrient use efficiency and grain yield. Additionally, grain yield increased with increasing nutrient uptake.

### 3.7. Crop Recovery and Agronomic Efficiency of Applied Nitrogen

The crop recovery and agronomic efficiency of the applied N in both pot and field trials were determined (Tables 12 and 13). The results showed that the crop recovery of applied N (RE_N_) in the pot trial was higher with the soils amended with biochar than in the normal practice. Additionally, the RE_N_ increased with decreasing N fertilizer rate (Table 12). This indicates that biochar in the treatments with N fertilizer enhanced N availability more than the rice plant requirement as compared to the plants under the normal N fertilization. This might be due to limitation in the amount of N the plants can absorb within a given period besides the fact that the chicken litter biochar had some amount of N. Crop recovery of applied N (RE_N_) of the field trial was indifferent from RE_N_ in the pot trial except for T6 and T7 where RE_N_ declined (Table 13). Additionally, the agronomic efficiency of the applied N (AE_N_) was not different from RE_N_ in both trials. However, the AE_N_ did not decline as compared to RE_N_ of the field trial (Table 13).

## 4. Conclusions

Coapplication of chicken litter biochar and urea can increase soil nutrient availability, nutrient use efficiency, dry matter yield, crop recovery, and agronomic efficiency in rice cultivation. Urea and K application was also reduced by 25% and 62.5%, respectively, whereas Egypt rock phosphate, magnesium oxide, and chelated ZnCoBor were 100% reduced in both pot and field studies. The grain yield in T5 was increased to 7.556 t ha^−1^ which is 44.36% higher and significantly different from yield of T2 (4.206 t ha^−1^). Additionally, biochar and the N rates (100%, 75%, 50%, 25%, and 0%) in T4, T5, T6, T7, and T8, respectively, stimulated the availability of other nutrients, especially P and K in the pot and field studies. There is a significant relationship between internal nutrient use efficiency and grain yield. Additionally, grain yield increased with increasing nutrient uptake. Finally, it is essential to look at the relationship between nutrient uptake and grain yield of rice instead of concentrating on only fertilization and grain yield as demonstrated in this study.

## Figures and Tables

**(a):** 

Macronutrients	
pH	8.5
%	
Total carbon	63.7
Fixed carbon	61.2
Nitrogen	2.8
Phosphate	2.6
Potassium	3.9
Calcium	5.9
Sulphur	0.59
Ash content	23.7

**(b):** 

Micronutrients	
Av. particle size	0.5–2 mm
mg kg^−1^	
Silicon	2.3
Aluminium	1.5
Potassium oxide	16.3
Boron	62
Copper	167
Manganese	1130
Zinc	856
Magnesium oxide	6.7
Arsenic	2.1
Cadmium	0.7
Chromium	9.6
Mercury	0.06
Nickel	14
Lead	12

Source: Black Earth Company in north of Bendigo, Victoria, Australia.

**Table 2:** Fertilization schedule recommended by Muda Agricultural Development Authority, 2013, and the equivalent rates used in the pot trial.

Local rice variety MR219, 105 to 111 days to maturity				
Plant growth stages	Early tillering growth	Active growth	Formation of stalk	Grain filling
Days after transplanting	15 to 20	35 to 40	50 to 55	70 to 75

Fertilizer type	Mixture fertilizers (Government aid)	Urea (Government aid)	Additional substance of fertilizer 12:12:17:2MgO + TE	Additional substance of fertilizer 12:12:17:2MgO + TE

Application rates (kg ha^−1^)	360 kg ha^−1^	100 kg ha^−1^ (1 bag alcove^−1^)	175 kg ha^−1^	175 kg ha^−1^

The mixture fertilizers (Government aid) = 17.5N : 15.5P_2_O_5_ : 10K_2_O.

**Table 3:** Biochar rates and fertilization schedule of the pot study.

Plant growth stages		Early tillering growth	Active growth	Formation of stalk	Grain filling
Days after transplanting		15 to 20	35 to 40	50 to 55	70 to 75
Treatments	Biochar rates	g hill^−1^			
T1	0	0	0	0	0
T2	0	Mix A1	0.4	Mix B1	Mix B1
T3	20	Mix A1	0.4	Mix B1	Mix B1
T4	20	0.55 urea only	1.2	0.18 urea only	0.18 urea only
T5	20	0.41 urea only	0.9	0.14 urea only	0.14 urea only
T6	20	0.28 urea only	0.6	0.09 urea only	0.09 urea only
T7	20	0.14 urea only	0.3	0.05 urea only	0.05 urea only
T8	20	0	0	0	0

Mix A1 = (0.55 g urea + 0.50 g TSP + 0.24 g MOP).

Mix B1 = (0.18 g urea + 0.19 g TSP + 0.20 g MOP + 0.014 MgO).

**Table 4:** Biochar rate and fertilization schedule of the field study.

Plant growth stages		Early tillering growth	Active growth	Formation of stalk	Grain filling
Days after transplanting		15 to 20	35 to 40	50 to 55	70 to 75
Treatments	Biochar rates	g plot^−1^			
T1	0	0	0	0	0
T2	0	Mix A2	40	Mix B1	Mix B1
T4	2000	55 urea only	40	18 urea only	18 urea only
T5	2000	40.3 urea only	30	14 urea only	14 urea only
T6	2000	27.5 urea only	20	9 urea only	9 urea only
T7	2000	13.8 urea only	10	5 urea only	5 urea only
T8	2000	0	0	0	0

Mix A2 = (55 g urea + 50 g TSP + 24 g MOP).

Mix B2 = (18.3 g urea + 18.7 g TSP + 19.8 g MOP + 1.4 MgO).

**Table 5:** Effects of biochar and nitrogen fertilization on soil chemical properties in the pot study.

Treatment	T1	T2	T3	T4	T5	T6	T7	T8
pHw	5.3^b^ ± 0.10	5.2^b^ ± 0.07	5.6^a^ ± 0.06	5.8^a^ ± 0.07	5.8^a^ ± 0.06	5.9^a^ ± 0.08	5.8^a^ ± 0.01	5.7^a^ ± 0.09

	%							
Organic matter	9.80^c^ ± 0.48	17.0^b^ ± 1.47	20.5^ab^ ± 1.04	20.0^ab^ ± 1.00	19.5^ab^ ± 0.29	22.0^a^ ± 1.08	18.8^ab^ ± 1.03	19.3^ab^ ± 1.03
Total carbon	1.13^c^ ± 0.06	1.97^b^ ± 0.17	2.38^ab^ ± 0.12	2.32^ab^ ± 0.12	2.26^ab^ ± 0.03	2.55^a^ ± 0.13	2.18^ab^ ± 0.12	2.23^ab^ ± 0.12
Total N	0.077^a^ ± 0.007	0.056^a^ ± 0.004	0.063^a^ ± 0.007	0.084^a^ ± 0.006	0.070^a^ ± 0.008	0.070^a^ ± 0.008	0.084^a^ ± 0.016	0.077^a^ ± 0.007

	mg kg^−1^							
Available NO^3−^	1.05^c^ ± 0.20	1.93^abc^ ± 0.34	2.10^abc^ ± 0.29	2.45^a^ ± 0.45	2.10^abc^ ± 0.29	2.28^ab^ ± 0.18	1.75^abc^ ± 0.20	1.23^bc^ ± 0.18
Exchangeable NH_4_^+^	1.75^c^ ± 0.20	8.76^a^ ± 0.45	9.81^a^ ± 0.70	10.33^a^ ± 0.44	10.05^a^ ± 0.44	10.51^a^ ± 0.40	10.16^a^ ± 0.35	4.55^b^ ± 0.45
Exchangeable P	3.75^c^ ± 0.24	7.21^c^ ± 0.77	19.6^ab^ ± 0.92	18.56^ab^ ± 0.96	17.07^b^ ± 4.01	18.23^ab^ ± 0.77	17.59^ab^ ± 0.53	21.26^a^ ± 1.40
Total P	70.08^d^ ± 0.65	116.65^c^ ± 8.78	225.45^a^ ± 16.34	187^b^ ± 5.73	187.65^b^ ± 4.19	202.44^ab^ ± 5.75	214.34^b^ ± 29.05	183.15^b^ ± 19.42

	cmol kg^−1^							
CEC	6.60^ab^ ± 0.15	6.35^b^ ± 0.07	7.90^a^ ± 0.30	7.45^ab^ ± 0.44	6.95^ab^ ± 0.26	6.18^b^ ± 0.11	7.33^ab^ ± 0.30	7.13^ab^ ± 44
Exchangeable Acidity	0.68^a^ ± 0.027	0.68^a^ ± 0.006	0.37^b^ ± 0.014	0.35^bc^ ± 0.016	0.34^bc^ ± 0.005	0.32^bc^ ± 0.008	0.31^bc^ ± 0.020	0.29^c^ ± 0.011
Exchangeable Al^3+^	0.53^a^ ± 0.013	0.53^a^ ± 0.009	0.22^b^ ± 0.009	0.22^b^ ± 0.006	0.24^b^ ± 0.015	0.24^b^ ± 0.009	0.22^b^ ± 0.010	0.20^b^ ± 0.008
Exchangeable H^+^	0.15^ab^ ± 0.023	0.18^a^ ± 0.013	0.15^ab^ ± 0.014	0.13^ab^ ± 0.020	0.09^b^ ± 0.012	0.09^b^ ± 0.014	0.09^b^ ± 0.012	0.09^b^ ± 0.015
Total K^+^	2.06^a^ ± 0.32	1.45^a^ ± 0.14	1.57^a^ ± 0.26	1.38^a^ ± 0.12	1.58^a^ ± 0.14	1.61^a^ ± 0.33	1.28^a^ ± 0.25	1.34^a^ ± 0.12
Exchangeable K^+^	0.30^ab^ ± 0.03	0.18^b^ ± 0.05	0.31^ab^ ± 0.02	0.28^ab^ ± 0.05	0.31^ab^ ± 0.02	0.37^a^ ± 0.05	0.21^b^ ± 0.03	0.27^ab^ ± 0.03
Exchangeable Cu^2+^	0.050f ± 0.0016	0.060^e^ ± 0.0028	0.082^c^ ± 0.0018	0.089^c^ ± 0.007	0.097^b^ ± 0.0023	0.103^ab^ ± 0.0012	0.110^a^ ± 0.0013	0.072^a^ ± 0.0026
Exchangeable Mn^2+^	0.15^ab^ ± 0.014	0.13^ab^ ± 0.004	0.17^ab^ ± 0.002	0.13^ab^ ± 0.027	0.13^ab^ ± 0.036	0.08^ab^ ± 0.031	0.15^ab^ ± 0.019	0.18^a^ ± 0.003
Exchangeable Fe^2+^	0.38^ab^ ± 0.10	0.53^a^ ± 0.04	0.37^ab^ ± 0.03	0.30^ab^ ± 0.08	0.30^b^ ± 0.08	0.18^b^ ± 0.03	0.26^b^ ± 0.04	0.35^b^ ± 0.01
Exchangeable Zn^2+^	0.006^ab^ ± 0.0003	0.005^ab^ ± 0.0003	0.008^ab^ ± 0.0009	0.007^ab^ ± 0.0006	0.006^ab^ ± 0.0010	0.007^ab^ ± 0.0010	0.009^a^ ± 0.0017	0.004^b^ ± 0.0006
Exchangeable Na^+^	6.28^a^ ± 2.07	3.42^a^ ± 0.90	6.46^a^ ± 0.97	5.64^a^ ± 3.35	2.60^a^ ± 0.10	6.91^a^ ± 2.10	6.88^a^ ± 1.98	4.64^a^ ± 1.98
Exchangeable Ca^2+^	17.59^b^ ± 2.27	20.35^b^ ± 1.49	32.74^a^ ± 1.38	29.16^ab^ ± 1.43	26.69^ab^ ± 3.13	25.16^ab^ ± 4.02	28.89^ab^ ± 2.91	27.03^ab^ ± 1.25
Exchangeable Mg^2+^	6.67^a^ ± 0.86	4.08^b^ ± 0.24	6.75^a^ ± 0.44	5.45^ab^ ± 0.21	4.05^b^ ± 0.52	3.97^b^ ± 0.28	3.29^b^ ± 0.51	7.57^a^ ± 0.59

Different letters within a row indicate significant difference between means of four replicates ± standard error using Tukey's test at *P* ≤ 0.05.

**Table 6:** Effects of biochar and nitrogen fertilization on soil chemical properties in the field study.

Treatment	T1	T2	T4	T5	T6	T7	T8	Before
pHw	4.6^a^ ± 0.16	4.6^a^ ± 0.20	4.9^a^ ± 0.20	4.9^a^ ± 0.19	5.0^a^ ± 0.14	4.7^a^ ± 0.17	4.5^a^ ± 0.11	4.9^a^ ± 0.04

	%							
Organic matter	7.5^c^ ± 0.65	8.3^c^ ± 1.38	15.3^a^ ± 1.55	13.0^a^ ± 0.95	14.3^ab^ ± 0.91	13.3^a^ ± 0.25	14.5^a^ ± 0.95	10.8^bc^ ± 0.85
Total carbon	0.87^c^ ± 0.07	0.96^c^ ± 0.16	1.77^a^ ± 0.18	1.51^a^ ± 0.11	1.65^b^ ± 0.11	1.54^a^ ± 0.03	1.68^a^ ± 0.11	1.25^bc^ ± 0.10
Total N	0.04^b^ ± 0.008	0.05^b^ ± 0.013	0.11^a^ ± 0.013	0.11^a^ ± 0.013	0.09^ab^ ± 0.013	0.09^ab^ ± 0.007	0.07^b^ ± 0.008	0.05^b^ ± 0.007

	mg kg^−1^							
Available NO_3_^−^	0.88^a^ ± 0.18	1.05^a^ ± 0.20	2.10^a^ ± 0.29	1.75^a^ ± 0.20	1.75^a^ ± 0.20	1.93^a^ ± 0.34	1.40^a^ ± 0.29	1.05^a^ ± 0.35
Exchangeable NH_4_^+^	1.93^d^ ± 0.18	3.33^d^ ± 0.34	3.68^cd^ ± 0.18	2.80^a^ ± 0.08	2.98^bc^ ± 0.15	2.98^abc^ ± 0.18	2.28^ab^ ± 0.22	1.58^abc^ ± 0.18
Available P	1.14^a^ ± 0.18	2.64^a^ ± 0.21	3.60^a^ ± 0.54	4.88^a^ ± 0.82	4.03^a^ ± 0.74	2.64^a^ ± 0.49	3.34^a^ ± 2.08	2.84^a^ ± 0.64
Total P	68.03^a^ ± 12.99	77.35^a^ ± 6.39	77.06^a^ ± 7.97	86.22^a^ ± 8.08	74.02^a^ ± 5.63	83.05^a^ ± 8.31	76.94^a^ ± 2.29	64.80^a^ ± 4.64

	cmol kg^−1^							
Total K	2.89^a^ ± 0.56	2.28^a^ ± 0.17	1.76^a^ ± 0.28	2.22^a^ ± 0.81	1.92^a^ ± 0.14	2.87^a^ ± 1.12	2.65^a^ ± 0.80	3.30^a^ ± 1.14
Available K	0.40^d^ ± 0.05	1.07^ab^ ± 0.05	0.71^bc^ ± 0.05	0.75^bc^ ± 0.03	0.99^ab^ ± 0.18	0.66^cd^ ± 0.06	0.37^d^ ± 0.06	1.01^ab^ ± 0.12
CEC	2.88^c^ ± 0.18	2.75^c^ ± 0.36	6.65^ab^ ± 0.68	5.93^ab^ ± 0.22	5.55^ab^ ± 0.54	7.25^a^ ± 0.48	6.50^ab^ ± 0.59	4.58^bc^ ± 0.10
Exchangeable Acidity	1.10^ab^ ± 0.04	1.06^abc^ ± 0.13	0.73^cd^ ± 0.05	0.74^cd^ ± 0.05	0.55^d^ ± 0.04	0.79^bcd^ ± 0.09	0.68^d^ ± 0.06	1.32^a^ ± 0.07
Exchangeable Al^3+^	0.91^a^ ± 0.06	0.71^b^ ± 0.07	0.43^d^ ± 0.04	0.40^d^ ± 0.01	0.36^d^ ± 0.03	0.52^d^ ± 0.04	0.49^bc^ ± 0.03	1.24^cd^ ± 0.05
Exchangeable H^+^	0.19^a^ ± 0.07	0.35^a^ ± 0.06	0.30^a^ ± 0.08	0.34^a^ ± 0.06	0.19^a^ ± 0.03	0.27^a^ ± 0.05	0.19^a^ ± 0.07	0.08^a^ ± 0.02
Exchangeable Cu^2+^	0.0076^a^ ± 0.0004	0.0069^b^ ± 0.0002	0.0069^b^ ± 0.0009	0.0060^ab^ ± 0.0005	0.0052^ab^ ± 0.0010	0.0032^ab^ ± 0.0001	0.0067^b^ ± 0.0002	0.0119^ab^ ± 0.0006
Exchangeable Mn^2+^	0.44^a^ ± 0.06	0.39^a^ ± 0.07	0.37^a^ ± 0.05	0.37^a^ ± 0.06	0.40^a^ ± 0.04	0.56^a^ ± 0.05	0.33^a^ ± 0.07	0.27^a^ ± 0.07
Exchangeable Fe^2+^	1.88^b^ ± 0.08	1.82^a^ ± 0.07	0.15^b^ ± 0.10	0.04^b^ ± 0.01	0.06^b^ ± 0.01	0.05^b^ ± 0.02	0.08^a^ ± 0.01	0.16^b^ ± 0.01
Exchangeable Zn^2+^	0.0017^a^ ± 0.0004	0.0018^b^ ± 0.0009	0.0011^ab^ ± 0.0003	0.0009^b^ ± 0.0002	0.0023^b^ ± 0.0011	0.0018^ab^ ± 0.0012	0.0037^b^ ± 0.0019	0.0068^b^ ± 0.0008
Exchangeable Na^+^	5.21^a^ ± 0.34	4.77^a^ ± 0.21	4.49^a^ ± 0.09	4.43^a^ ± 0.08	4.68^a^ ± 0.18	4.32^a^ ± 0.15	4.87^a^ ± 0.63	5.25^a^ ± 0.39
Exchangeable Ca^2+^	17.70^a^ ± 1.37	18.12^b^ ± 1.23	19.41^b^ ± 1.17	19.82^ab^ ± 0.70	18.96^ab^ ± 1.93	19.90^ab^ ± 0.47	16.89^b^ ± 1.85	26.39^ab^ ± 2.76
Exchangeable Mg^2+^	14.79^b^ ± 0.28	12.22^a^ ± 2.01	11.23^a^ ± 1.30	11.42^ab^ ± 0.56	12.14^ab^ ± 1.48	15.55^a^ ± 1.31	13.23^a^ ± 2.25	5.27^a^ ± 0.93

Different letters within a row indicate significant difference between means of four replicates ± standard error using Tukey's test at *P* ≤ 0.05.

**Table 7:** Effects of biochar and nitrogen fertilization on measured variables of rice plants in the pot study.

Treatment	T1	T2	T3	T4	T5	T6	T7	T8
Plant height (cm)	76.20^d^ ± 1.17	86.48^c^ ± 0.48	98.38^a^ ± 1.13	98.08^a^ ± 0.79	94.80^ab^ ± 0.76	93.15^b^ ± 0.46	88.05^c^ ± 1.02	80.05^d^ ± 1.07

	hill^−1^							
Number of tillers	6.0^e^ ± 0.10	12^c^ ± 0.29	15^a^ ± 0.25	16^a^ ± 0.29	13^b^ ± 0.48	13^bc^ ± 0.41	12^c^ ± 0.29	10^d^ ± 0.25
Number of leaves	8^f^ ± 1.44	66^c^ ± 1.29	81^a^ ± 0.85	78^ab^ ± 0.65	73^ab^ ± 1.25	75^b^ ± 0.65	57^c^ ± 1.38	38^e^ ± 0.65

	g hill^−1^							
Root dry weight	3.11^d^ ± 0.26	10.63^bc^ ± 0.57	14.55^a^ ± 0.40	11.58^b^ ± 0.08	11.74^b^ ± 0.07	10.94^b^ ± 0.04	9.28^c^ ± 0.22	9.24^c^ ± 0.60
Dry matter yield	5.86^c^ ± 0.51	23.15^c^ ± 0.84	33.81^a^ ± 0.32	33.55^a^ ± 0.50	31.57^a^ ± 0.75	27.37^b^ ± 1.06	23.54^c^ ± 0.21	16.31^d^ ± 0.38

Different letters within a row indicate significant difference between means of four replicates ± standard error using Tukey's test at *P* ≤ 0.05.

**Table 8:** Effects of biochar and nitrogen fertilization on measured variables of rice plants in the field study.

Treatment	T1	T2	T4	T5	T6	T7	T8
Plant height (cm)	72.5^c^ ± 2.77	93.0^ab^ ± 1.42	101.8^a^ ± 0.81	100.5^ab^ ± 0.83	95.2^ab^ ± 1.43	90.7^b^ ± 1.34	73.0^c^ ± 3.84
Culm height (cm)	58.9^c^ ± 2.34	75.8^b^ ± 1.03	80.8^a^ ± 0.77	81.2^a^ ± 1.67	79.4^a^ ± 1.51	73.7^a^ ± 0.59	58.0^a^ ± 4.46
Number of tillers per 0.04 m^2^	8^b^ ± 0.30	12^ab^ ± 0.49	16^a^ ± 0.73	15^a^ ± 0.29	12^ab^ ± 0.40	12^ab^ ± 0.34	10^b^ ± 0.56
Number of leaves per 0.04 m^2^	8^f^ ± 0.25	66^c^ ± 0.78	81^a^ ± 2.74	78^ab^ ± 0.50	73^ab^ ± 0.64	75^b^ ± 0.73	38^e^ ± 1.06
Number of panicles per 0.04 m^2^	7^d^ ± 0.14	11^b^ ± 0.18	13^a^ ± 0.41	13^a^ ± 0.45	10^bc^ ± 0.52	9^c^ ± 0.74	6^d^ ± 0.16
Dry matter yield (g per 0.04 m^2^)	5.6^e^ ± 0.60	17.0^bc^ ± 1.98	27.7^a^ ± 2.24	23.2^ab^ ± 1.09	19.7^b^ ± 0.99	13.1^cd^ ± 1.34	7.5^de^ ± 1.51
Total grain per panicle	110^bc^ ± 11.64	104^c^ ± 2.19	136^abc^ ± 2.21	160^a^ ± 9.57	133^abc^ ± 6.43	151^ab^ ± 17.08	143^abc^ ± 15.28
% total grain filling per panicle	65.92^a^ ± 1.81	63.30^a^ ± 2.01	66.91^a^ ± 1.02	61.46^a^ ± 1.93	66.99^a^ ± 2.36	67.56^a^ ± 1.34	68.51^a^ ± 1.26
Yield (t ha^−1^)	2.612^d^ ± 0.27	4.206^cd^ ± 0.19	6.794^ab^ ± 0.25	7.559^a^ ± 0.43	5.233^bc^ ± 0.48	5.184^bc^ ± 0.66	3.429^d^ ± 0.42

Different letters within a row indicate significant difference between means of four replicates ± standard error using Tukey's test at *P* ≤ 0.05.

**Table 9:** Effects of coapplication of biochar and urea on nutrients uptake in a pot study.

Treatment	T1	T2	T4	T5	T6	T7	T8
	mg hill^−1^						
Total N	11.2^e^ ± 1.07	39.0^ab^ ± 2.33	43.7^a^ ± 2.72	43.7^a^ ± 1.24	34.4^bc^ ± 1.02	29.1^cd^ ± 1.71	21.9^d^ ± 0.54
Si	20.8^c^ ± 5.10	110.6^abc^ ± 9.05	219.7^a^ ± 21.43	166.7^ab^ ± 45.77	143.8^abc^ ± 40.35	150.1^abc^ ± 34.99	100.0^abc^ ± 5.62

	mg hill^−1^						
Total P	3.3^d^ ± 0.53	30.7^b^ ± 2.62	52.3^a^ ± 1.38	30.2^b^ ± 3.18	25.6^bc^ ± 0.81	22.0^bc^ ± 2.02	19.7^c^ ± 1.18
K^+^	46.3^e^ ± 8.20	238.4^d^ ± 11.01	506.0^a^ ± 52.28	347.3^bc^ ± 16.57	421.8^ab^ ± 13.96	383.0^b^ ± 15.97	361.8^bc^ ± 6.56
Ca^2+^	19.7^d^ ± 2.03	84.6^bc^ ± 10.30	147.8^a^ ± 11.07	137.3^a^ ± 7.73	142.0^a^ ± 8.13	126.3^a^ ± 4.29	119.4^ab^ ± 8.35
Mg^2+^	11.2^d^ ± 2.42	40.2^c^ ± 2.25	80.3^a^ ± 7.54	65.2^ab^ ± 4.01	83.6^a^ ± 10.91	67.2^ab^ ± 3.59	55.7^bc^ ± 1.54
Na^2+^	12.0^e^ ± 1.98	30.3^bcd^ ± 4.96	43.5^ab^ ± 5.37	35.4^abc^ ± 1.84	48.7^a^ ± 1.76	38.3^abc^ ± 2.33	25.4^cde^ ± 1.84
Fe^2+^	3.7^cd^ ± 0.59	16.7^a^ ± 0.71	6.2^b^ ± 0.58	4.4^bcd^ ± 0.32	4.9^bc^ ± 0.61	3.6^cd^ ± 0.51	2.5^d^ ± 0.22
Cu^2+^	0.05^d^ ± 0.005	0.25^c^ ± 0.019	0.45^ab^ ± 0.013	0.48^a^ ± 0.017	0.48^a^ ± 0.016	0.47^ab^ ± 0.023	0.38^b^ ± 0.017
Zn^2+^	0.9^b^ ± 0.28	4.6^ab^ ± 0.65	6.5^ab^ ± 2.88	8.4^a^ ± 0.91	6.7^ab^ ± 1.74	6.5^ab^ ± 1.71	6.8^ab^ ± 0.58
Mn^2+^	0.96^e^ ± 0.08	3.43^c^ ± 0.16	5.43^a^ ± 0.18	4.71^ab^ ± 0.09	5.12^a^ ± 0.26	3.96^bc^ ± 0.30	3.74^c^ ± 0.09

Different letters within a row indicate significant difference between means using Tukey's test at *P* ≤ 0.05.

**Table 10:** Effects of coapplication of biochar and urea on nutrients uptake in a field study.

Treatment	T1	T2	T4	T5	T6	T7	T8
	mg/0.04 m^2^						
Total N	1.90^d^ ± 0.05	11.61^cd^ ± 2.60	31.63^a^ ± 7.33	27.50^ab^ ± 1.55	17.18^bc^ ± 2.30	7.27^cd^ ± 0.90	3.31^cd^ ± 0.64
Si	41.4^b^ ± 2.28	58.4^ab^ ± 4.52	95.8^a^ ± 12.14	52.8^ab^ ± 8.12	63.2^ab^ ± 13.47	32.2^ab^ ± 2.62	44.6^b^ ± 12.26

	mg/0.04 m^2^						
Total P	2.73^e^ ± 0.10	9.14^de^ ± 1.23	38.32^a^ ± 2.34	31.44^ab^ ± 3.92	25.58^bc^ ± 2.81	16.91^cd^ ± 2.53	8.31^de^ ± 1.71
K^+^	119.0^d^ ± 11.19	306.5^c^ ± 36.96	439.1^ab^ ± 26.39	445.6^a^ ± 31.15	424.9^abc^ ± 14.26	307.6^bc^ ± 33.12	159.2^d^ ± 27.23
Ca^2+^	36.4^bc^ ± 2.12	48.6^bc^ ± 5.27	117.9^a^ ± 8.39	129.9^a^ ± 12.19	85.0^ab^ ± 6.35	49.3^bc^ ± 4.25	25.7^c^ ± 6.06
Mg^2+^	11.9^c^ ± 1.69	36.0^c^ ± 4.76	99.5^a^ ± 9.53	93.7^ab^ ± 5.31	71.1^b^ ± 8.00	36.4^c^ ± 4.00	19.4^c^ ± 4.90
Na^2+^	116.2^ab^ ± 14.74	144.6^ab^ ± 35.21	256.3^ab^ ± 86.48	227.0^a^ ± 34.03	41.5^ab^ ± 7.75	98.6^b^ ± 30.37	91.6^ab^ ± 31.85
Fe^2+^	1.29^c^ ± 0.16	4.64^bc^ ± 0.80	9.80^a^ ± 1.52	7.86^ab^ ± 1.22	5.27^bc^ ± 0.63	3.10^c^ ± 0.58	1.39^c^ ± 0.24
Cu^2+^	0.012^a^ ± 0.004	0.018^a^ ± 0.005	0.023^a^ ± 0.011	0.034^a^ ± 0.007	0.018^a^ ± 0.007	0.008^a^ ± 0.005	0.013^a^ ± 0.007
Zn^2+^	0.29^c^ ± 0.06	0.94^ab^ ± 0.20	1.35^a^ ± 0.12	1.36^a^ ± 0.19	1.18^a^ ± 0.05	0.87^abc^ ± 0.10	0.42^bc^ ± 0.10
Mn^2+^	0.011^a^ ± 0.002	0.039^a^ ± 0.005	0.071^a^ ± 0.026	0.092^a^ ± 0.024	0.124^a^ ± 0.048	0.056^a^ ± 0.017	0.047^a^ ± 0.014

Different letters within a row indicate significant difference between means using Tukey's test at *P* ≤ 0.05.

**Table 11:** Correlation among nitrogen fertilization, N, P, and K uptake, and grain yield.

	N applied	N uptake	P uptake	K uptake
N applied				

N uptake	0.796^*∗*^ 0.032			

P uptake	0.635 0.126	0.949^*∗*^ 0.001		

K uptake	0.771^*∗*^ 0.042	0.900^*∗*^ 0.006	0.913^*∗*^ 0.004	

Yield	0.671 0.099	0.919^*∗*^ 0.003	0.936^*∗*^ 0.002	0.919^*∗*^ 0.003

^*∗*^Significant correlation at *P* ≤ 0.05.

**Table 12:** Effects of nitrogen application on crop recovery and agronomic efficiency under pot trial.

Treatment	Total N applied	Plants N uptake	Dry matter yield (DMY)	Crop recovery efficiency of applied N (RE_N_)	Agronomic efficiency of applied N (AE_N_)
	g hill^−1^				
T1	0	0.08	5.86	—	—
T2	282.50	0.20	23.15	0.0004	0.06
T3	282.50	0.22	33.81	0.0005	0.10
T4	282.50	0.20	33.55	0.0004	0.10
T5	211.80	0.19	31.57	0.0005	0.12
T6	141.30	0.16	27.37	0.0006	0.15
T7	70.65	0.13	23.54	0.0007	0.25
T8	0	0.08	16.31	—	—

**Table 13:** Effects of nitrogen application on crop recovery and agronomic efficiency under field trial.

Treatment	Total N applied	Plants N uptake	Yield	Crop recovery efficiency of applied N (REN)	Agronomic efficiency of applied N (AEN )
	g/0.04 m^2^				
T1	0	0.02	1.05	—	—
T2	327.5	0.12	1.68	0.00031	0.0020
T4	327.5	0.32	2.72	0.00092	0.0051
T5	245.63	0.28	3.02	0.00106	0.0081
T6	163.75	0.17	2.09	0.00092	0.0064
T7	81.88	0.07	2.07	0.00061	0.0126
T8	0	0.03	1.37	—	—
